# Expression quantitative trait loci in sheep liver and muscle contribute to variations in meat traits

**DOI:** 10.1186/s12711-021-00602-9

**Published:** 2021-01-18

**Authors:** Zehu Yuan, Bolormaa Sunduimijid, Ruidong Xiang, Ralph Behrendt, Matthew I. Knight, Brett A. Mason, Coralie M. Reich, Claire Prowse-Wilkins, Christy J. Vander Jagt, Amanda J. Chamberlain, Iona M. MacLeod, Fadi Li, Xiangpeng Yue, Hans D. Daetwyler

**Affiliations:** 1 State Key Laboratory of Grassland Agro-ecosystems; Key Laboratory of Grassland Livestock Industry Innovation, Ministry of Agriculture and Rural Affairs; Grassland Agriculture Engineering Center, Ministry of Education; College of Pastoral Agriculture Science and Technology, Lanzhou University, Lanzhou, 730020 People’s Republic of China; 2 Agriculture Victoria, AgriBio, Centre for AgriBioscience, Bundoora, VIC 3083 Australia; 3 Institutes of Agricultural Science and Technology Development (Joint International Research Laboratory of Agriculture & Agri-Product Safety), Yangzhou University, Yangzhou, 225000 People’s Republic of China; 4 Faculty of Veterinary & Agricultural Science, University of Melbourne, Parkville, VIC 3010 Australia; 5 Agriculture Victoria, Hamilton Centre, Hamilton, VIC 3300 Australia; 6 School of Applied Systems Biology, La Trobe University, Bundoora, VIC 3083 Australia

## Abstract

**Background:**

Variants that regulate transcription, such as expression quantitative trait loci (eQTL), have shown enrichment in genome-wide association studies (GWAS) for mammalian complex traits. However, no study has reported eQTL in sheep, although it is an important agricultural species for which many GWAS of complex meat traits have been conducted. Using RNA sequence data produced from liver and muscle from 149 sheep and imputed whole-genome single nucleotide polymorphisms (SNPs), our aim was to dissect the genetic architecture of the transcriptome by associating sheep genotypes with three major molecular phenotypes including gene expression (geQTL), exon expression (eeQTL) and RNA splicing (sQTL). We also examined these three types of eQTL for their enrichment in GWAS of multi-meat traits and fatty acid profiles.

**Results:**

Whereas a relatively small number of molecular phenotypes were significantly heritable (h^2^ > 0, *P *< 0.05), their mean heritability ranged from 0.67 to 0.73 for liver and from 0.71 to 0.77 for muscle. Association analysis between molecular phenotypes and SNPs within ± 1 Mb identified many significant cis-eQTL (false discovery rate, FDR < 0.01). The median distance between the eQTL and transcription start sites (TSS) ranged from 68 to 153 kb across the three eQTL types. The number of common variants between geQTL, eeQTL and sQTL within each tissue, and the number of common variants between liver and muscle within each eQTL type were all significantly (*P* < 0.05) larger than expected by chance. The identified eQTL were significantly (*P *< 0.05) enriched in GWAS hits associated with 56 carcass traits and fatty acid profiles. For example, several geQTL in muscle mapped to the *FAM184B* gene, hundreds of sQTL in liver and muscle mapped to the *CAST* gene, and hundreds of sQTL in liver mapped to the *C6* gene. These three genes are associated with body composition or fatty acid profiles.

**Conclusions:**

We detected a large number of significant eQTL and found that the overlap of variants between eQTL types and tissues was prevalent. Many eQTL were also QTL for meat traits. Our study fills a gap in the knowledge on the regulatory variants and their role in complex traits for the sheep model.

## Background

Gene expression varies between tissues and individuals [1, 2], and their phenotypes can be shaped by gene expression [3]. Gene and exon expression levels can be directly quantified by counting the RNA sequence (RNA-seq) reads that map to the gene and its exons [4, 5]. RNA-splicing can be estimated by isoform ratios, exon inclusion levels [6] and intron excision ratios [7]. There are statistical challenges to estimate isoform abundance [8] and technical effects [7] to estimate exon inclusion levels when using conventional short-read data. Therefore, calculating the excised intron ratios could be an accurate method to quantify RNA-splicing, because excised introns are inferred directly from short reads that span exon–exon junctions [9]. Thus, the three types of molecular phenotypes, i.e. gene expression, exon expression and intron excision ratio, can be accurately quantified via RNA-seq.

Different DNA mutations among individuals can result in varied molecular phenotypes in a population. A mutation that regulates nearby (± 1 Mb) molecular phenotypes from RNA-seq data (i.e. gene expression, exon expression and intron excision ratio) is defined as a cis expression quantitative trait locus or eQTL(eQTL are defined here as gene expression QTL (geQTL), exon expression QTL (eeQTL), and splicing QTL (sQTL)). In beef cattle, geQTL have been investigated in liver and muscle [10–12]. Some of the geQTL that have been identified in humans, cattle, and pigs overlap with (or colocalize at) single nucleotide polymorphisms (SNPs) that are linked with diseases and complex traits [3, 13–16]. For example, ten QTL linked with pork meat quality co-localized with 12 eQTL in the *longissimus dorsi* muscle [16]. Therefore, eQTL analysis may improve our knowledge on the genetic architecture of complex traits. In dairy cattle, Xiang et al. [12] showed that eeQTL overlapped with geQTL and sQTL. Consequently, the combined analysis of geQTL, eeQTL and sQTL may increase the chance to identify loci that regulate gene expression [12, 17]. However, to our knowledge, eQTL have not been analyzed in sheep, although it is one of the major farm species for human consumption.

Sheep and lamb meat consumption continues to grow worldwide due to the animal’s adaptability to be farmed in different climatic conditions and to its unique culinary value [18]. Meat production and quality traits are polygenic and are influenced by many QTL, each making a small contribution to a trait [19, 20]. The polygenicity of sheep meat traits is supported by recent genome-wide association studies (GWAS) with SNPs [21–27]. In particular, Bolormaa et al. [28] used high-density SNPs (~ 500 K) to identify pleiotropic SNPs that are associated with 56 carcass composition traits, and Rovadoscki et al. [29] used 50 K SNPs to identify SNPs that are linked with fatty acid profiles [29]. The results from GWAS have laid the foundation for studying sheep meat characteristics that are regulated by genetic variants. However, the biological mechanisms that underlie how these QTL affect meat traits are not well known. In this study, we use eQTL data to begin to explore these mechanisms.

Using imputed whole-genome SNPs and RNA-seq data, our aim was to quantify the heritability of three molecular phenotypes and identify cis eQTL in sheep liver and muscle. Then, we investigated if the same SNPs were significantly associated with multiple eQTL phenotypes and/or if the eQTL were significant in both tissues. We also investigated if eQTL were enriched in GWAS QTL of multiple sheep meat traits [28, 29]. Our results contribute to a better understanding of how genomic differences in sheep lead to variation in complex meat traits.

## Methods

An overview of the analysis is shown in Additional file 1: Figure S1.

### Sample collection and RNA extraction

In total, 149 crossbred wether lambs (ewes: Merino $\times$ Border Leicester and Maternal/Coopworth Composites, nine sires: Polled Dorset and White Suffolk) between 7 and 8 months old were randomly selected for RNA-seq analysis from 436 male lambs that were balanced across dam and lamb nutritional treatments, birth types, breeds and sires [30]. Lambs were slaughtered in their experimental blocks on three slaughter dates [30]. Liver and *longissimus dorsi* muscle were sampled within 10 min from slaughter at the abattoir and were flash-frozen in liquid nitrogen and stored at − 80°C until use. Frozen liver and muscle tissues were ground using the Geno/Grinder^®^ 2010 (SPEX™, Metuchen, NJ, US). Ground tissue was homogenized in Trizol^®^ (Life Technologies™) and RNA extracted using the Trizol^®^ plus RNA extraction kit (Life Technologies™) according to the manufacturer’s instructions. RNA quality and integrity were evaluated by the 2100 Bioanalyzer (Agilent Technologies, Waldbronn, Germany). All samples with an RNA integrity number larger than 7 and a 28S/18S ratio higher than 1.0 were used for library preparation using the SureSelect Strand Specific RNA Library Prep Kit (Agilent Technologies).

### RNA-seq and data quality control

Libraries were randomly pooled and sequenced on a HiSeq2000 genome analyzer (Illumina Inc) in a paired end 100 cycle run to produce 20 million paired-reads per library. CASAVA v1.8 (Illumina Inc) was used to call fastq files. QuadTrim (https://bitbucket.org/arobinson/quadtrim) was used to trim adapter and low-quality bases (quality score < 20) from each end of the reads, and then to discard low-quality reads (failed chastity filter, or mean quality score < 20, or Ns > 3, or final length < 50). Finally, only paired-reads that passed the quality control criteria were kept for downstream analysis.

### Quantification of molecular phenotypes

Clean paired-reads were aligned to the sheep reference genome Oar_v3.1 (ftp://ftp.ensembl.org/pub/release-91/fasta/ovis_aries/dna/) using STAR [31] along with the annotation file (Ovis_aries.Oar_v3.1.91.gtf.gz, containing 27,054 genes). The parameters used for alignment are documented in Additional file 2: Table S1. Parameters were chosen to maximize uniquely mapped reads [32]. Each Sequence Alignment Map (SAM) file was sorted and transformed into a Binary Alignment Map (BAM) file by SAMtools [33]. The proportion of reads that were uniquely mapped was summarized from STAR alignment results (see Additional file 4: Table S2). Gene saturation was assessed as the number of detected genes per percent of total reads at increasing read depths, with reads sampled from each BAM file. Saturation of splice junctions and 3′/5′ bias were investigated using RseQC [34].

Read counts of each gene and each exon were calculated by FeatureCounts [5]. In each tissue, genes and exons with a count per million (CPM) higher than 1 in more than 30 individuals (> 20%) were retained, and log10(x + 1) transformed. Intron excision ratios were quantified by LeafCutter [7] as the change in intron usage in the intron cluster of which it is a member, where several intron clusters are often inferred within a gene. Intron excision ratios higher than 0 in at least 30 animals (> 20%) and mean ratio values higher than 0.01 across all individuals were retained and log10(x + 1) transformed, intron-wise quantile normalized and individual-wise Z-score standardized [12].

### Estimation of the heritability of molecular phenotypes

In total, 13,243 genes, 63,872 exons and 91,699 intron excision events in liver and 12,989 genes, 60,230 exons and 87,257 intron excision events in muscle were used to estimate heritabilities. Each molecular phenotype was adjusted for fixed effects, including slaughter day, feed lot pen number and replicate, dam breed, sire breed, birth type and dam gestational body condition score, using the lm() function in R (https://www.r-project.org/). Heritabilities ($h^{2}$) of molecular phenotypes, which are defined here as the proportion of the phenotypic variance explained by all the SNPs, were estimated using the ASReml^®^ software [35] by fitting the following linear mixed model:

$${\mathbf{y}} = {\mathbf{1}}_{\varvec{n}} \mu + {\mathbf{a}} + {\mathbf{e}},$$

where ${\mathbf{y}}$ is an ${\text{n }} \times 1$ vector of the molecular phenotypes of all individuals (${\text{n}}$ = 149), ${\mathbf{1}}_{{\mathbf{n}}}$ is a vector of 1s, $\mu$ is the overall mean, ${\mathbf{a}}$ is an ${\text{n }} \times 1$ vector of additive genetic effects, following a normal distribution ${\text{N }}\sim \left( {0, {\mathbf{G}}\sigma_{a}^{2} } \right)$, where ${\mathbf{G}}$ is the genomic relationship matrix (GRM) [36] calculated from the ~ 500 K SNP panel for 149 individuals, $\sigma_{a}^{2}$ is additive genetic variance, ${\mathbf{e}}$ is a ${\text{n }} \times 1$ vector of random residuals, following a normal distribution ${\text{N }}\sim \left( {0, {\mathbf{I}}\sigma_{e}^{2} } \right)$. The heritability of molecular phenotypes was calculated as:

$$h^{2} = \frac{{\sigma_{a}^{2} }}{{\sigma_{a}^{2} + \sigma_{e}^{2} }}.$$

The differences in heritability between the three molecular phenotypes within a tissue were tested by the wilcox.test() function in R.

### Imputed whole-genome SNPs

All 149 individuals were genotyped with the high-density Ovine SNP Beadchip (HD, ~ 500 K SNP) and imputed to whole-genome sequence genotypes as part of a study by Bolormaa et al. [37]. Briefly, 935 animals with whole-genome sequence variants (117 pure Merino, 726 European breeds, 92 other breeds) were selected as the reference population (https://www.ebi.ac.uk/eva/?eva-study=PRJEB31241) to impute the target population (~ 47,000). Both reference and target population HD genotypes were phased using the Eagle software with default parameters and the target population genotypes were imputed to whole-genome sequence using the Minmac3 software with default parameters [38, 39]. Finally, SNPs with an R^2^ (imputation quality index reported from Minimac3) lower than 0.4 were removed to reduce the impact of poorly imputed SNPs. On average, the empirical imputation accuracy across all target breeds was 0.97. Imputed variants in the 149 individuals with a minor allele frequency (MAF) lower than 0.05 were removed to avoid spurious associations in the subsequent analyses.

### Cis eQTL detection

Molecular phenotypes were tested for association with all the SNPs within ± 1 Mb of each molecular feature (to reduce computational burden). In total, 12,373 genes, 56,233 exons, and 79,146 intron excision events in liver and 12,151 genes, 53,660 exons and 76,824 intron excision events in muscle located on autosomes were used for eQTL mapping. Association testing between each molecular phenotype and SNPs was implemented one SNP at a time using the Wombat software [40] by fitting the following linear mixed model:

$${\mathbf{y}} = {\mathbf{1}}_{\varvec{n}} \mu + {\mathbf{Z}}\beta + {\mathbf{a}} + {\mathbf{e}},$$

where ${\mathbf{y}}$ is a ${\text{n }} \times 1$ vector of the molecular phenotypes of all individuals (${\text{n}}$ = 149 in the current study), ${\mathbf{Z}}$ is a ${\text{n }} \times 1$ vector of SNP genotypes coded as 0, 1 or 2, $\beta$ is marker effect, ${\mathbf{a}}$ is a ${\text{n }} \times 1$ vector of polygenic effects, following a normal distribution ${\text{N }}\sim \left( {0, {\mathbf{G}}\sigma_{a}^{2} } \right)$, ${\mathbf{e}}$ is a ${\text{n }} \times 1$ vector of random residuals, following a normal distribution ${\text{N }}\sim \left( {0, {\mathbf{I}}\sigma_{e}^{2} } \right)$. For each molecular phenotype, variance components estimated from ASreml^®^ were regarded as prior information for Wombat to implement association testing. When a SNP was significantly associated with multiple molecular phenotypes, only the most significant one was retained. Finally, SNPs with a false discovery rate (FDR) lower than 0.01 were regarded as significant cis eQTL.

Transcription start site (TSS) coordinates of all genes were obtained from Ensembl via BioMart (http://www.ensembl.org). The absolute distance between a cis eQTL and the TSS of the eQTL anchored gene was calculated. The eQTL functional catalogues were determined using the NGS-SNP pipeline [41] with the Variant Effect Predictor software [42].

### Relationships between eQTL type and their effect across tissues

It is possible that the three eQTL types are not independent. geQTL are associated with a change in total gene expression, which can affect all the isoforms or only one of them. In addition, eeQTL are associated with a change in exon expression, which can affect all the exons in a gene, and therefore total gene expression, or only one exon and therefore a single isoform. Thus, it is likely that some geQTL are also eeQTL or sQTL or both. For this reason, we tested the overlap between the three eQTL types, i.e. we tested whether a single variant was significantly associated with two molecular phenotypes. In addition, it is possible that the same eQTL has an effect in both tissue types. Thus, we tested the overlap between tissues for each eQTL type, i.e. we tested whether a single variant was significantly associated with a particular molecular phenotype in both tissues.

The significance (p-values) of overlaps between two sets (e.g. set ${\text{A}}$ and set ${\text{B}}$) were calculated with the hypergeometric enrichment test R function phyper, which requires the number of elements in the background set ${\text{W}}$ ($N, {\text{A}} \subseteq {\text{W}}, {\text{B}} \subseteq {\text{W}}$), the number of elements in set ${\text{A}}$ ($n$), the number of elements in set ${\text{B}}$ ($M$), and the number of elements in the intersection of ${\text{A}}$ and ${\text{B}}$ ($m, {\text{A}} \cap {\text{B}}$). Equivalently, one can use the newGeneOverlap and testGeneOverlap functions in the GeneOverlap R package [43]. When testing for significance of overlaps between different eQTL types within and across tissues, $N$ is the number of SNPs used for eQTL identification ($N$ = 20,824,844 in this study). For overlaps of geQTL between different tissues, $N$ is the number of expressed genes in both liver and muscle. Similarly, to test the overlap of eeQTL and sQTL between tissues, $N$ is the number of expressed exons and intron excision events in both liver and muscle, respectively.

### Enrichment of eQTL in GWAS hit regions

Because the GWAS of Bolormaa et al. [28] and Rovadoscki et al. [25] included many detailed post-slaughter phenotypes in sheep, we used their results to investigate the overlap with the eQTL identified in our study. However, based on the difference in SNP densities in these studies (Bolormaa et al. [28] (~ 500 K) and Rovadoscki et al. [29]) (~ 50 K) and in our analysis (~ 20 million), it was unlikely that eQTL would overlap exactly with GWAS SNPs. Thus, we investigated the overlap of eQTL in a defined interval of 25 kb up- and down-stream of significant (FDR < 0.01) pleiotropic SNPs (all 932 significant pleiotropic SNPs from the multi-trait meta-analysis in Bolormaa et al. [28] and significant regions in Rovadoscki et al. [29]). The OvineSNP50 BeadChip mean gap size between probes was 50.9 kb (median = 42.6 kb), thus the use of 25-kb intervals should capture most of the eQTL in these regions. The hypergeometric test (Eq. 1) was used to investigate whether identified eQTL were significantly enriched in GWAS hit regions, where ${\text{N}}$ is the number of all SNPs used in the eQTL analysis (${\text{N}}$ = 20,824,844 in this study); ${\text{n}}$ is the number of significant eQTL; ${\text{M}}$ is the number of SNPs in the current study that are located in GWAS hit regions and m is the number of significant eQTL in ${\text{M}}$.

## Results

### Data quality

In total, 20,824,844 SNPs passed quality control and were used for eQTL association testing. The distribution of these SNPs is shown in Additional file 3: Figure S2A. After filtering the low-quality raw reads, 8379 million clean read pairs from 298 samples were retained, i.e. an average of 27.76 million read pairs per sample (see Additional file 4: Table S2). Roughly, 7107 million clean read pairs were uniquely mapped to the sheep genome, i.e. an average of 23.85 million read pairs per sample (see Additional file 4: Table S2). The gene body plots of all expressed genes indicated little 5′ bias (see Additional file 3: Figure S2B). In the RNA-seq technology, saturation is reached when an increment in the number of reads does not result in the detection of additional expressed genes or in the calling of more features, e.g., splice junctions [44]. Our gene saturation analysis showed that the number of detected genes increased with increasing percent of total reads and that, when the percent of reads reached 75% of the total reads, few additional genes were detected [see Additional file 3: Figure S2C]. This result indicates that most of the expressed genes were detected by the RNA-seq study. The splice junction saturation analysis revealed that when the percent of reads reached 100%, the number of all detected splice junctions (including annotated and novel splice junctions) did not reach an asymptote (see Additional file 3: Figure S2D). Almost all annotated splice junctions were rediscovered (see Additional file 3: Figure S2E), but novel splice junctions did not reach an asymptote (see Additional file 3: Figure S2F). This result suggests that some novel splice junctions were still missing at this level of coverage.

### Heritability of molecular phenotypes

Molecular phenotypes with a heritability ($h$^2^) higher than 0 (Z test, *P *< 0.05) were considered heritable. In liver, the mean heritabilities across the 290 gene expression (2.19%), 7014 exon expression (10.98%) and 2389 intron excision ratios (2.61%) of heritable molecular phenotypes were 0.73, 0.67 and 0.71, respectively (Table 1), and in muscle, the mean heritabilities across the 487 of gene expression (3.75%), 12,599 exon expression (20.92%) and 4198 intron excision ratios (4.81%) of heritable molecular phenotypes were 0.77, 0.73 and 0.71, respectively. Both in liver and muscle, the level of heritable exon expression was higher than that of gene expression and intron excision ratio.

**Table 1:** Summarized information for molecular phenotype heritability

Molecular phenotype	Total number	Number of heritable phenotypes	Heritable ratio	Mean heritability (standard deviation)
Liver gene expression	13,243	290	0.0219	0.73 (0.14)
Liver exon expression	63,872	7014	0.1098	0.67 (0.13)
Liver intron excision	91,699	2389	0.0261	0.72 (0.14)
Muscle gene expression	12,989	487	0.0375	0.77 (0.12)
Muscle exon expression	60,230	12,599	0.2092	0.73 (0.13)
Muscle intron excision	87,257	4198	0.0481	0.71 (0.13)

Details include the total number of molecular phenotypes (total number), the number of molecular phenotypes that were heritable (number of heritable phenotypes, heritability > 0, P < 0.05), the ratio of heritable molecular phenotypes to total number (heritable ratio) and the mean heritability of the heritable molecular phenotypes (mean heritability) and standard deviations

In liver, 640,976 (FDR < 0.01) geQTL, 376,181 eeQTL and 678,657 sQTL were identified (Table 2) and (see Additional file 5: Figure S3A). The geQTL, eeQTL and sQTL in liver were associated with 3631, 2265 and 2633 genes, respectively (Table 2). In muscle, 356,380 geQTL, 223,900 eeQTL and 383,044 sQTL were identified (Table 2) and (see Additional file 5: Figure S3A). The geQTL, eeQTL and sQTL in muscle were associated with 2396, 1564 and 2047 genes, respectively (Table 2). The number of significant eQTL was larger than the number of tagged genes, which indicated that many SNPs were in high linkage disequilibrium (LD) or that there were multiple variants associated with the same gene (Table 2).

**Table 2:** Summary of detected expression quantitative trait loci (eQTL), including gene expression QTL, (geQTL), exon expression QTL, (eeQTL), and splicing QTL, (sQTL) including number of QTL detected, number of genes in which those QTL were located and number of eQTL per gene

Tissue	Cis eQTL type	eQTL number	Gene number	eQTL per gene
Liver	geQTL	640,976	3631	176.53
	eeQTL	376,181	2265	166.08
	sQTL	678,657	2633	257.75
Muscle	geQTL	356,380	2396	148.74
	eeQTL	223,900	1564	143.16
	sQTL	383,044	2047	187.12

**Fig. 1:**
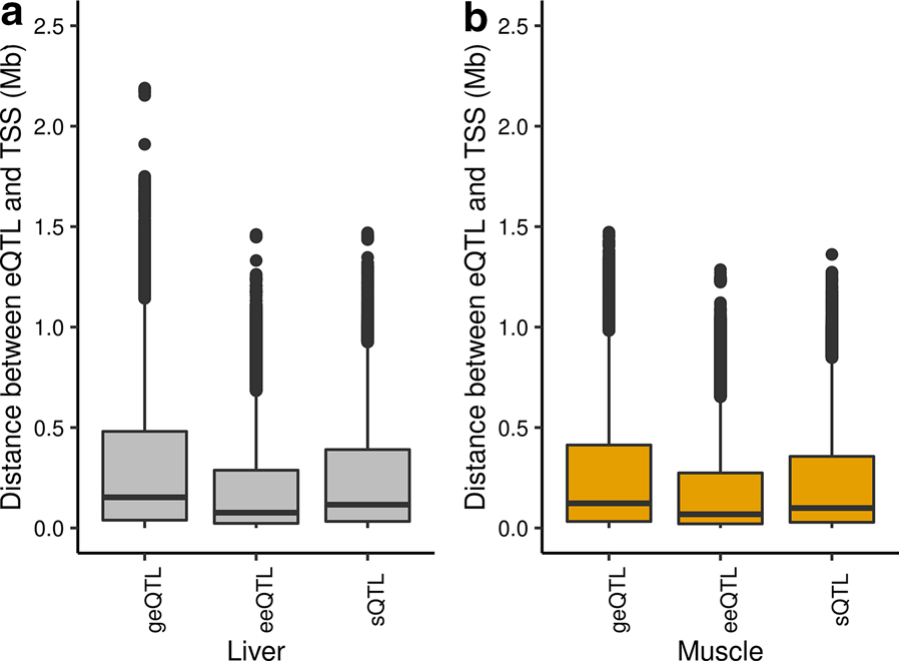
Absolute distance between expression quantitative trait loci (eQTL, which include gene expression QTL, geQTL; exon expression QTL, eeQTL and splicing QTL, sQTL) and gene transcription start sites (TSS) in liver (**a**) and muscle (**b**)

In liver, the median distances between the geQTL, eeQTL, and sQTL and TSS were 153, 76 and 116 kb, respectively (Fig. 1a), and in muscle, they were 123, 68 and 99 kb, respectively (Fig. 1b). Both in liver and muscle, eeQTL were closer to TSS than geQTL or sQTL.

Compared to the total number of SNPs in the genome, a decreased proportion of intergenic SNPs and an increased proportion of intronic, missense, synonymous, gene-end, UTR and splicing SNPs were found for the three eQTL types (Table 3). Whereas we expected sQTL to be enriched in the annotated “Splice” category, followed by eeQTL, and geQTL [12], we found that that all three eQTL types shared a similar proportion of ‘Splice’ variants both in liver and muscle. However, the absolute numbers of ‘Splice’ sQTL, eeQTL and geQTL are in the expected order.

**Table 3:** Proportional functional annotation of expression quantitative trait loci (eQTL, including gene expression QTL, geQTL; exon expression QTL, eeQTL and splicing QTL, sQTL) and all single nucleotide polymorphisms (SNPs) used for eQTL detection

Functional category	All	Liver			Muscle		
		geQTL	eeQTL	sQTL	geQTL	eeQTL	sQTL
Splice	0.0007	0.0016	0.0021	0.0019	0.0018	0.0026	0.0022
UTR	0.0022	0.0057	0.0076	0.0060	0.0069	0.0081	0.0072
Gene_end	0.0606	0.1219	0.1371	0.1226	0.1316	0.1477	0.1340
Synonymous_variant	0.0032	0.0075	0.0102	0.0085	0.0085	0.0124	0.0096
Missense_variant	0.0018	0.0047	0.0057	0.0052	0.0047	0.0060	0.0056
Intron_variant	0.3032	0.4327	0.4540	0.4434	0.4356	0.4700	0.4658
Intergenic	0.6271	0.4249	0.3822	0.4113	0.4095	0.3520	0.3746
Others	0.0010	0.0011	0.0010	0.0012	0.0015	0.0013	0.0011

All eQTL were annotated as intergenic, intronic, missense, synonymous, up or downstream (Gene_end), in untranslated regions (UTR, 3′ or 5′), having some splicing function (Splice) or other (all other categories)

### Relationship between eQTL types and their effect across tissues

**Fig. 2:**
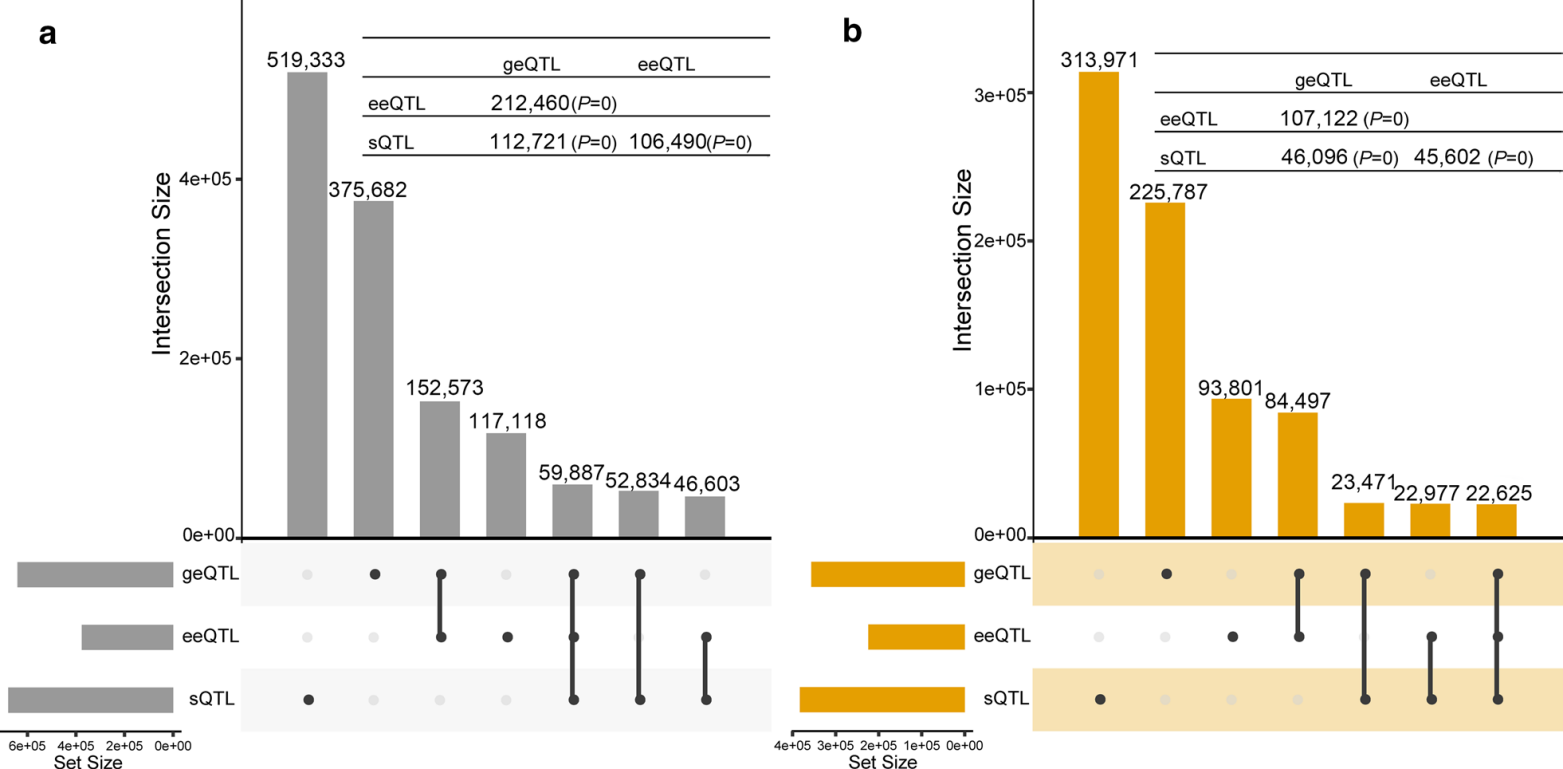
Overlap between the three types of expression quantitative trait loci (eQTL, which include gene expression QTL, geQTL; exon expression QTL, eeQTL and splicing QTL, sQTL) in liver (**a**) and muscle (**b**). Table in the top right part shows pair-wise the number of common eQTL and P-values. The numbers in the bottom left part denote the number of significant eQTL for each type. Dots denote the eQTL types. Connection lines connecting the dots show eQTL types included in the comparison. The number above each bar shows the number of eQTL for each type (column 1 to column 3) or shared (column 4 to column 7). UpSet Plot was plotted by UpSetR R package (https://cran.r-project.org/web/packages/UpSetR/)

The amount of overlap of SNPs between the three eQTL types within each tissue was significantly (*P* < 0.05) larger than expected by chance (Fig. 2). The largest number of shared SNPs was observed between geQTL and eeQTL (Fig. 2), followed by that between geQTL and sQTL, and between eeQTL and sQTL.

A significant (*P* < 0.05) overlap of geQTL, eeQTL and sQTL between liver and muscle was observed (see Additional file 6: Figure S4a). The largest number of overlapping SNPs between liver and muscle was observed for sQTL, followed by eeQTL, and geQTL (see Additional file 6: Figure S4a). Genes, exons and intron excision events tagged by eQTL were shared at a significant level between liver and muscle (see Additional file 6: Figure S4B). Interestingly, when eQTL were significant in both liver and muscle, their effect (t-value) was usually in the same direction (see Additional file 6: Figure S4c), with eeQTL being the most consistent.

**Fig. 3:**
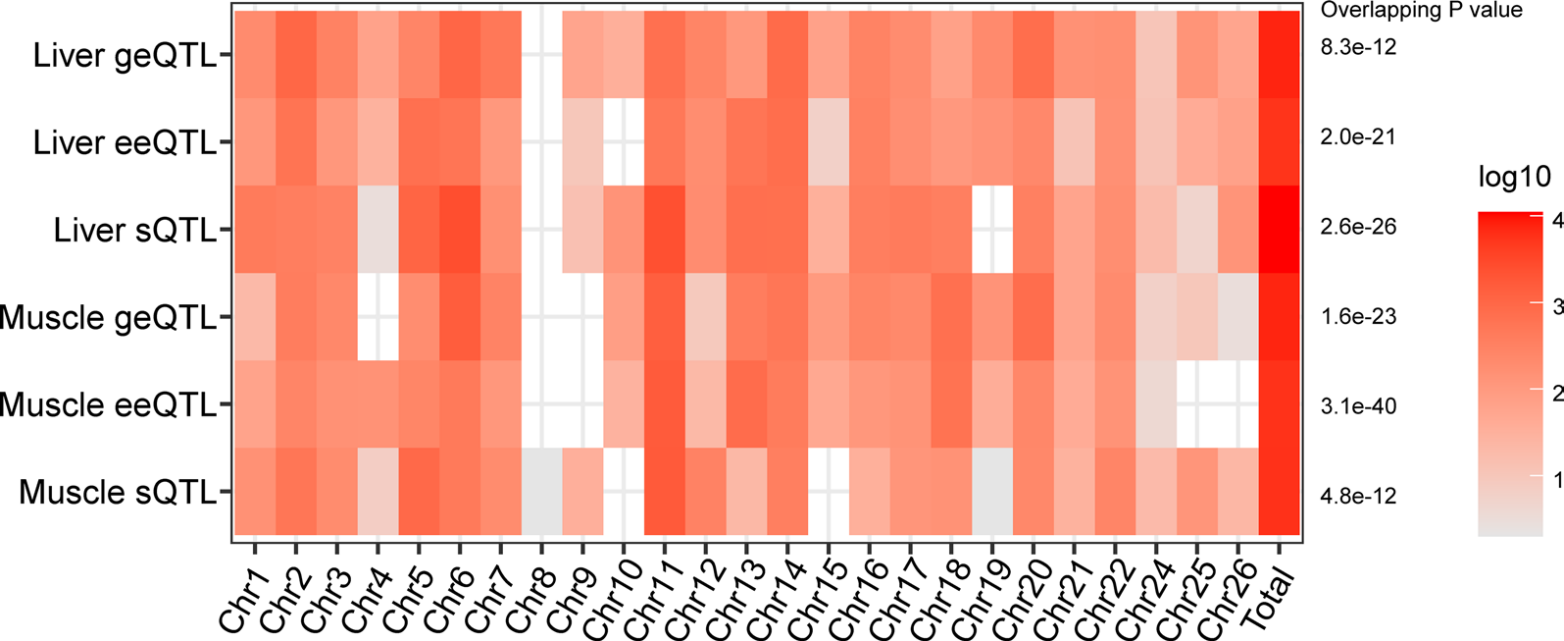
Number of expression quantitative trait loci (eQTL, which include gene expression QTL, geQTL; exon expression QTL, eeQTL and splicing QTL, sQTL) that overlap with genome-wide association study (GWAS) hit regions linked with body composition [28]. The scale of red color represents the number of shared pleiotropic single nucleotide polymorphisms (SNPs) between eQTL and GWAS hit regions

We used the results from two published GWAS [28] and [25] for meat traits to evaluate the significant regions and eQTL in our study. First, 1130 pleiotropic SNP regions (from a multi-trait meta-analysis, FDR < 0.01) [28] were used to assess the overlap between these and eQTL. A summary of the results is shown in Fig. 3 and the detailed results are documented in Additional file 7: Table S3. All three types of eQTL in liver and muscle were significantly enriched in GWAS hit regions (*P* < 0.05, Fig. 3). In total, 43.45% (491/1130), 26.99% (305/1130) and 52.12% (589/1130) of the GWAS hit regions identified by Bolormaa et al. [28] were covered by geQTL, eeQTL and sQTL in liver, respectively, and 43.98% (497/1130), 26.02% (294/1130) and 30.62% (346/1130) of the GWAS hit regions were covered by geQTL, eeQTL and sQTL in muscle, respectively.

Second, we used 27 significant regions linked with fatty acid profiles [29] to investigate their overlap with eQTL from our study. Generally, liver eQTL were more enriched in genomic regions linked with fatty acid profile than muscle eQTL (Table 4). The identified geQTL in liver were significantly (*P* < 0.05) enriched in genomic regions that were associated with saturated fatty acids (SFA), polyunsaturated fatty acids (PUFA), the ratio of linoleic acid (ω6) to alpha-linolenic acid (ω3) and the ratio of PUFA to SFA, the identified eeQTL in liver were significantly (*P* < 0.05) enriched in genomic regions that are associated with PUFA, and the sQTL were significantly (*P* < 0.05) enriched in genomic regions that are associated with SFA, monounsaturated fatty acids (MUFA), and ω6/ω3 ratios. In muscle, geQTL were significantly (*P* < 0.05) enriched in genomic regions that are associated with SFA and MUFA. Both the identified eeQTL and sQTL in muscle were significantly (*P* < 0.05) enriched in genomic regions that are associated with SFA and PUFA.

**Table 4:** Expression quantitative trait loci (eQTL, which include gene expression QTL (geQTL), exon expression QTL (eeQTL), and splicing QTL (sQTL)) enriched in genome-wide association study (GWAS) hit regions linked with fatty acid profiles [29]

Fatty acid	Liver geQTL	Liver eeQTL	Liver sQTL	Muscle geQTL	Muscle eeQTL	Muscle sQTL
Saturated fatty acid (SFA)						
Myristic acid (C14:0)	*612 (1.2e*−*33)*	229 (0.15)	*707 (9e*−*51)*	*1255 (0e *+ *00)*	*1176 (0.00)*	233 (0.15)
Palmitic acid (C16:0)	*406 (2.6e*−*26)*	0 (1.00)	0 (1.00)	0 (1.00)	0 (1.00)	*340 (1.2e*−*48)*
Stearic acid (C18:0)	*296 (6.7e*−*03)*	5 (1.00)	*570 (5.4e*−*59)*	*997 (0.00)*	43 (1.00)	150 (0.6)
Total SFA	*617 (5.8e*−*08)*	3 (1.00)	*1978 (0.00)*	55 (1.00)	43 (1.00)	5 (1.00)
Monounsaturated fatty acids (MUFA)						
Palmitoleic acid (C16:1)	*764 (1.7e*−*13)*	9 (1.00)	*2031 (0.00)*	*947 (8.8e*−*178)*	61 (1.00)	147 (1.00)
Oleic acid (C18:1)	94 (1.00)	5 (1.00)	226 (1.00)	61 (1.00)	36 (1.00)	56 (1.00)
Total MUFA	221 (1.00)	3 (1.00)	*1505 (7.8e*−*152)*	32 (1.00)	36 (1.00)	0 (1.00)
Polyunsaturated fatty acids (PUFA)						
Linoleic acid (C18:2, ω6)	*396 (1.1e*−*51)*	0 (1.00)	0 (1.00)	0 (1.00)	0 (1.00)	0 (1.00)
alpha-Linolenic acid (C18:3, ω3)	*1172 (2.1e*−*116)*	*696 (9.6e*−*71)*	292 (1.00)	*818 (4.6e*−*128)*	*329 (2.1e*−*18)*	183 (1.00)
Conjugated linoleic acid (CLA, c9t11)	*277 (2e*−*17)*	96 (0.45)	142 (0.99)	88 (0.58)	6 (1.00)	*201 (3e*−*21)*
Total ω3	0 (1.00)	*197 (1.5e*−*03)*	127 (1.00)	0 (1.00)	0 (1.00)	65 (1.00)
Total ω6	*396 (1.6e*−*37)*	0 (1.00)	0 (1.00)	0 (1.00)	6 (1.00)	0 (1.00)
Total PUFA	*396 (6.7e*−*10)*	0 (1.00)	0 (1.00)	1 (1.00)	6 (1.00)	0 (1.00)
ω6/ω3 and PUFA/SFA ratios						
ω6/ω3	*418 (1.00)*	13 (1.00)	*1481 (4.4e*−*128)*	208 (1.00)	36 (1.00)	0 (1.00)
PUFA/SFA	*396 (3.6e*−*154)*	0 (1.00)	0 (1.00)	0 (1.00)	0 (1.00)	0 (1.00)
Total	6849			4610		

The number in each cell denotes the number of eQTL located in genomic regions linked with a fatty acid trait

The number in parentheses denotes the P-value for overlap between the two datasets

### Examples of QTL that may be eQTL

**Fig. 4:**
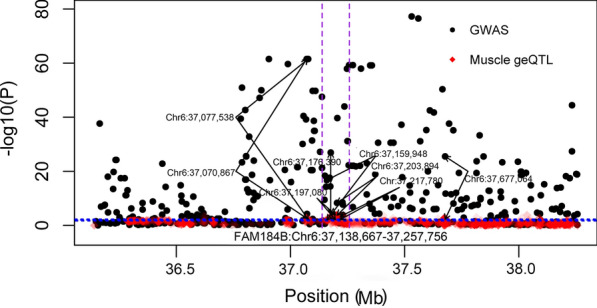
Gene expression quantitative trait loci (geQTL) in muscle associated with the *FAM184B* gene in red with the significant threshold denoted by the blue dotted line. Black dots are genome-wide association study (GWAS) for multi-traits [28]. Y-axis is the −log10P for both GWAS and geQTL. The vertical purple dotted lines denote the gene boundary

The *FAM184B* gene (*family with sequence similarity 184 member B*; Chr6:37138667–37257756) is located in a candidate QTL region (close to Chr6:37.53 Mb) for meat traits in sheep, which was identified by using the 50 K [27] and HD SNPs chips [28]. Eight SNPs (Chr6:37070867, Chr6:37077538, Chr6:37159948, Chr6:37176390, Chr6:37197080, Chr6:37203894, Chr6:37217780 and Chr6:37677064) that are located in close proximity to *FAM184B* and that have been identified as significant pleiotropic SNPs linked with meat traits [28], were also geQTL in muscle for *FAM184B* (Fig. 4) in our study.

**Fig. 5:**
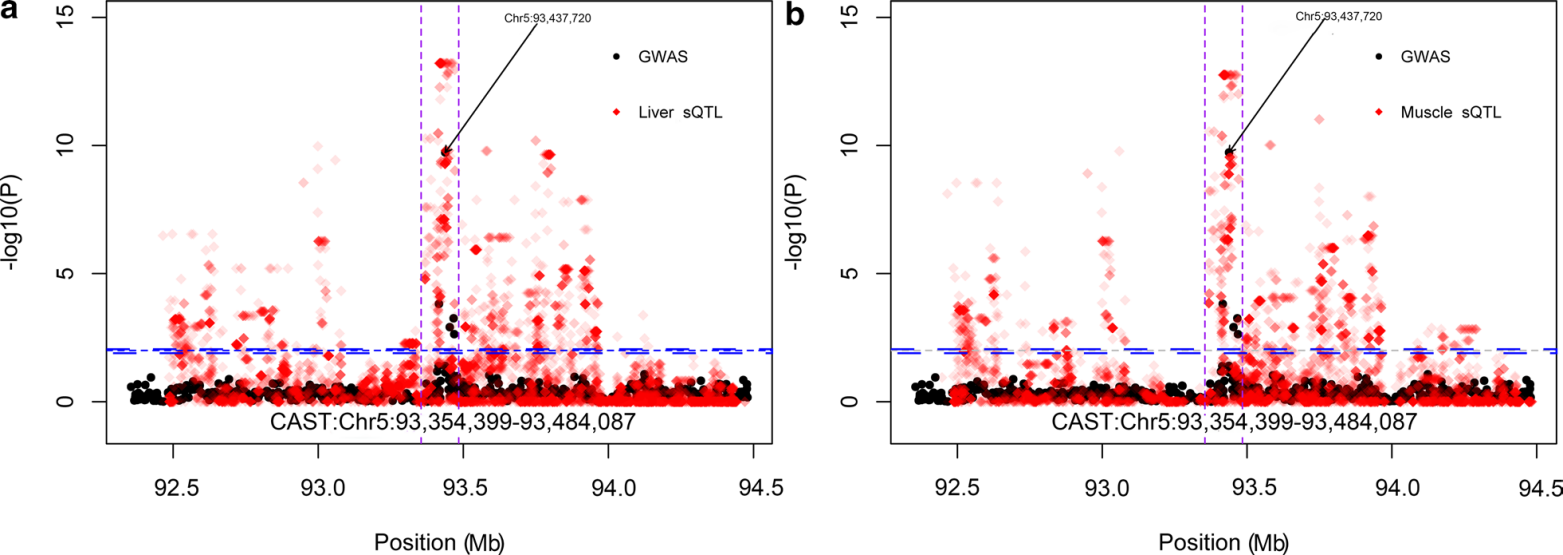
Splicing quantitative trait loci (sQTL) in liver (**a**) and muscle (**b**) mapped to the *CAST* gene in red, with the significant threshold indicated as the blue dotted line. Black dots are genome-wide association study (GWAS) for multi-trait [28]. Y-axis is the −log10P value. Vertical purple dotted lines denote the CAST gene boundary

**Fig. 6:**
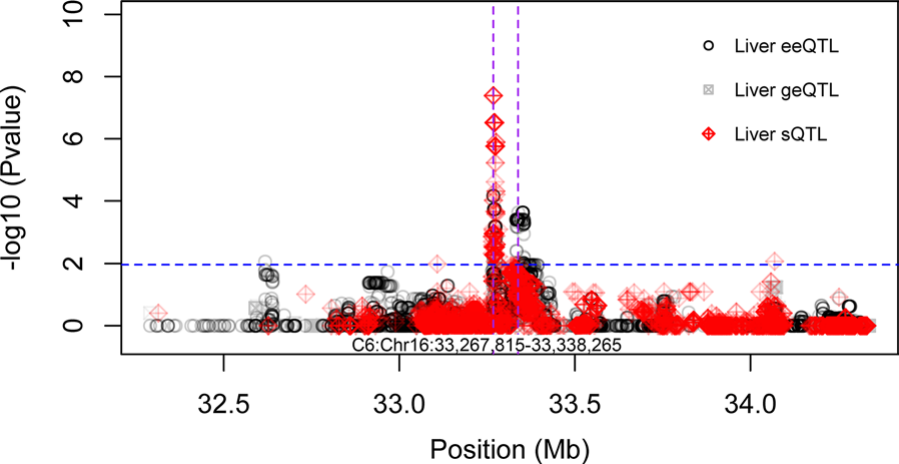
Expression quantitative trait loci (eQTL) in red associated with the *C6* gene in liver, the significant threshold is indicated by the blue dotted line.“geQTL, eeQTL, sQTL denote gene expression, exon expression and splicing QTL, respctively. Y-axis denotes the eQTL P-value. The vertical purple dotted lines denote the *C6* gene boundary

The most significant SNP (Chr5:93437720*)* associated with tenderness [28] was located in the *CAST* gene (*calpastatin*; Chr5:93354399–93484087) (Fig. 5). We found 1985 sQTL in liver associated with three intron excision events in *CAST* (Chr5:93439378–93444596 and Chr5: 93394527–93427992), and 1800 sQTL in muscle associated with intron excision events (Chr5:93435918-93437744, Chr5:93439378–93444596 and Chr5:93482729–93483763) in *CAST*. Some sQTL in liver and muscle were associated with the same intron excision region (Chr5:93439378–93444596), which was between exon 9 (Chr5:93439292–93439378) and exon 11 (Chr5:93444596–93444694) of *CAST* (Fig. 5). A putative QTL region (Chr16:33207525–33550464) associated with alpha-linolenic acid (ω3) [29] included the *C6* (*complement C6*, Chr16:33267815-33338265) gene. In total, 146 eeQTL and 128 sQTL in liver mapped to the *C6* gene QTL region (Fig. 6). The 146 eeQTL were associated with the expression of two exons in the *C6* gene (exon1, Chr16:33267815–33267879; and exon 18, Chr16:33338090–33338265). The 128 sQTL were associated with four intron excision regions (Chr16:33266238–33267815, Chr16:33266238–33272429, Chr16:33266695–33272429 and Chr16: 33267879–33272429), for which, the closely located exon (C6: Chr16:33267815–33267879, exon1) was regulated by an eeQTL.

## Discussion

Our study is the first that analyses the associations between sequence variants and cis molecular phenotype variation (± 1 Mb) at transcriptomic levels (including gene expression, exon expression and splicing) in sheep liver and muscle.

We estimated the heritability of global gene expression, exon expression, and intron excision events in sheep, using SNPs. Molecular phenotypes were considered heritable when they were significantly different from zero. In humans, the median SNP heritability of gene expression has been reported to range from 0.1 to 0.34 [45–51] for 31 to 85% of the heritable molecular phenotypes across tissues (including lymphoblastoid cell lines, lymphocytes, blood and adipose tissue) [45–47, 49, 51]. In pig, the reported mean SNP heritabilities of molecular phenotypes range from 0.18 to 0.51 [16, 52]. In the current study, the number of heritable molecular phenotypes was smaller than in most previous publications. However, on average, the non-zero heritability estimates of molecular phenotypes are high, and our estimation of the heritability of molecular phenotypes is higher than that reported in humans and pigs. In part this is due to our relatively small sample size, which will cause heritabilities to be overestimated. However, the transcriptome is also the first level at which DNA mutations have an impact, once properly captured, the molecular heritability is expected to be higher than the heritability of complex traits, which is impacted by DNA mutations via several omics-layers [53]. In addition, measuring gene expression based on RNA-seq data is quite precise, compared to gross measurements of phenotypic traits (e.g., body weight). This can reduce the error variance in some molecular phenotypes, which in part increases the signal-to-noise ratio in the estimation of molecular heritability.

We found that a relatively low proportion of molecular phenotypes reached a heritability that was significantly different from zero, which could be due to several reasons. First, whereas our study is the largest sheep eQTL study to date, its sample size (i.e. 149 lambs) was modest, which can lead to large variance errors for the heritability estimates. As such, many of the molecular phenotypes with a moderate heritability estimate would have not been deemed significant due to the large standard error. With a larger dataset, it is likely that the number of significant molecular phenotypes would increase and the mean heritability estimates would decrease as more molecular phenotypes with a moderate heritability reached significance. Second, gene expression is transient and often time-point- and tissue-specific, and so lower expression levels may lead to lower heritability estimates. We studied only two tissues in 149 sheep, and it would be interesting to study the molecular heritability of other sheep tissues in the future. Third, our dataset allowed us to test only SNP or additive heritability of molecular phenotypes, although regulatory relationships between SNPs and adjacent genes can be largely non-additive. Future studies using much larger sample sizes should test for non-additive effects to recapture the ‘missing’ heritability in gene expression.

This study only considered cis eQTL to reduce the number of tests [54]. A larger number of tests for trans eQTL would have required a more stringent threshold and likely would have reduced the power to detect cis eQTL. In addition, cis eQTL may be easier to detect because their effects are larger than those of trans eQTL [54]. The majority of the identified sheep eQTL were within 250 kb from TSS, which is consistent with the results from human studies [55]. The identified eeQTL in sheep were closer to TSS than the sQTL and geQTL, which disagrees with a study in cattle that reported that sQTL were closer to TSS than other types of eQTL [12] and that used a different and more stringent definition for sQTL. Indeed, they report only intron excision events that had an adjacent exon inclusion event, which is an exon usage phenotype, i.e. exon read count/gene read count [12]. In our study, all sQTL were intron excision events only. In human and cattle, sQTL have been reported to be enriched in intron variants [12, 55, 56] compared to all the SNPs detected. Consistently, in our study, eeQTL in liver and muscle had a higher proportion of intron SNPs than geQTL. However, in sheep liver and muscle, the identified sQTL did not have a higher proportion of intron SNPs than eeQTL. This inconsistent result could be explained by differences in the definition of significant sQTL, in computational methods, and in sample sizes, tissues and species between these studies.

In humans, significant commonalities of sQTL between tissues and of geQTL between tissues were reported [57, 58]. Recent studies have suggested that, in cattle, three types of eQTL (sQTL, eeQTL and geQTL) were shared between tissues [12] and that, in pig, geQTL were extensively shared between liver and muscle [59]. Our results support the extensive sharing of all eQTL types between liver and muscle, but disagree with other studies [47, 60]. The ratio of eQTL overlap between tissues depends on the similarity of biological functions, with more similar tissues sharing more eQTL [61]. Our results support the significant overlap of eQTL between liver and muscle [see Additional file 5: Figure S3], which could be due to the similarity of biological functions that regulate meat traits between liver and muscle.

A recent eQTL study in humans, which detected geQTL, eeQTL, and sQTL from blood samples, showed significant enrichment for GWAS SNPs for disease or complex traits [62]. Similarly, we found that the three types of eQTL in sheep liver and muscle were significantly enriched in GWAS hit regions that were previously identified by Bolormaa et al. [28]. Whereas two published cattle studies showed a relatively limited overlap between eQTL and single-trait GWAS SNPs [11, 54], we found a relatively high overlap between eQTL and multi-trait QTL. Several reasons might have contributed to our results. First, the pleiotropic SNPs from multi-trait GWAS (multi-trait meta-analysis) may be more informative than SNPs from single-trait GWAS [28, 63] and this is supported by the relatively few eQTL that we observed in several genomic regions linked with single traits (e.g. fatty acid profiles). In addition, we used GWAS hit regions instead of exact overlaps (SNP to SNP), which could increase the probability of overlap because eQTL may be in LD with QTL. This is in line with a cattle study published in 2019, which used eQTL SNPs to build functional GRM that explained a large amount of the genetic variance in 34 complex traits [64]. Moreover, the relationship between the phenotypes included in the GWAS and the tissues used for the eQTL analysis may affect the overlap between GWAS and eQTL. For example, a recent study in cattle suggested that 10 of 163 SNPs associated with stature were identified as geQTL in white blood cells [13]. However, SNPs associated with milk production and fertility did not overlap with geQTL identified in white blood cells [54]. These results show that we still have a very limited understanding of how eQTL and trait QTL relate, and thus, future research in this area is required.

We highlight a few examples of QTL that may also be eQTL. In sheep, *FAM184B* was located in the most significant GWAS region that is associated with body weight [27] and body composition traits [28]. In cattle, the expression level of *FAM184B* was suggested to be regulated by cis eQTL [54]. In our study, five significant pleiotropic SNPs located near *FAM184B* [28] also affected the total expression level of *FAM184B* significantly and confirmed the previously reported association.

Our second example highlights *CAST*. In sheep, the most significant SNP (Chr5:93437720) associated with shear force (i.e. meat tenderness) was within the *CAST* gene [28]. Twenty-one isoforms of the human *CAST* gene are included in RefSeq (https://www.ncbi.nlm.nih.gov/gene/831#), and similarly, several isoforms of bovine *CAST* [65] are found in RefSeq (https://www.ncbi.nlm.nih.gov/gene/281039). This evidence lends support to our finding of an intron excised region (i.e. exon Chr5:93439378-93444596) detected in both sheep liver and muscle that indicates alternative splicing (skipping exon 10), but this mechanism needs to be validated using molecular techniques. The expression level of exon 10 in *CAST* was low and was removed from the association analysis in our study, which provides additional support for this exon-skip alternative splicing. In humans, SNPs have been identified that regulate alternative splicing in *CAST* [66]. We found that many sQTL in sheep liver and muscle mapped to this intron excised region (Chr5:93439378-93444596) within *CAST*, but unlike a study in cattle [11], we found no significant geQTL for *CAST*. Taken together, these results suggest that SNPs that are linked with tenderness (shear force) in sheep might regulate the expression of *CAST* by excluding the 10th exon from the transcript, while not altering the total level of expression. Recent research in humans suggested that a splice variant (rs7724759) in *CAST* affected exon abundance instead of gene abundance [67], which supports our finding.

The $\omega$3 and $\omega$6 polyunsaturated fatty acid composition of sheep meat and lamb is important for human health [68] and depends on both the dietary intake of the animal and the cellular metabolism, which is often controlled by genetic polymorphisms [69]. One example of a gene that controls $\omega$3 and $\omega$6 metabolism in the cell is the *C6* gene. This gene is located in a putative QTL region associated with alpha-linolenic acid (C18:3 $\omega$3) and total $\omega$3 [29]. *C6* has been reported to be specifically highly expressed in sheep liver [70], which supports its potential role in the fatty acid profile of meat, because the liver plays an essential role in fatty acid synthesis. A protective effect of *C6* deficiency on the development of diet-induced atherosclerosis has been observed when *C6*-deficient rabbits were fed a cholesterol-rich diet for 14 weeks [71], which suggests that it might play a role in lipid metabolism. In our study, the 143 significant sQTL in which the intron excision event had a flanking exon were identified as eeQTL, which supports the reliability of intron excision region detection. Since many significant liver sQTL for *C6* were located in a QTL region that is linked with ω3 polyunsaturated fatty acid, it could be an eQTL for *C6* splicing level. Therefore, we cautiously speculate that SNPs, which regulate the content of $\omega$3 polyunsaturated fatty acid in meat, may do so by regulating *C6* alternative splicing in liver, which might affect cellular lipid metabolism.

As the first detailed analysis of sheep cis eQTL, our study has its limitations. Many factors affect the performance of fine mapping, e.g., the local LD structure and sample size [72]. The strong LD between closely located SNPs results in multiple SNPs appearing as significantly linked with a molecular phenotype when analyzing one SNP at a time. We used 149 lambs from nine sires, which means that the local LD may be relatively strong and leads to broad eQTL peaks. In our study, the sample size was relatively small, which reduces the power to detect eQTL [54], whereas in the current human eQTL studies sample sizes reach tens of thousands of individuals [73].

## Conclusions

A relatively small number of molecular phenotypes had a SNP heritability significantly different from zero, but many significant cis eQTL were detected. These were often associated with several eQTL types and were significant in both liver and muscle tissue. The identified eQTL were significantly enriched in previously reported GWAS regions for meat traits, for example several geQTL in muscle mapped to *FAM184B*, hundreds of sQTL in liver and muscle mapped to *CAST*, and hundreds of sQTL in liver mapped to *C6*.

## Supplementary information

**Additional file 1: Figure S1.** Overview of the analysis. In total, 298 RNA-seq data in liver and muscle from 149 crossbred male wether lambs were aligned to the sheep reference genome Oar_v3.1 (ftp://ftp.ensembl.org/pub/release-91/fasta/ovis_aries/dna/) using STAR along with the annotation file (Ovis_aries.Oar_v3.1.91.gtf.gz, containing 27,054 genes). Gene and exon expression levels were quantified by counting the reads of the gene and exon using FeatureCount. RNA-splicing was estimated by calculating intron excision ratio using Leafcutter. Heritability (h2) of the three molecular phenotypes (gene expression, exon expression and intron excision ratio) were estimated using ASreml^®^. Wombat software was used to identify cis expression quantitative trait loci (eQTL, which include gene expression QTL (geQTL); exon expression QTL (eeQTL) and splicing QTL(sQTL)) within 1 Mb of the gene, exon or intron excision event. We investigated the overlap between eQTL and two genome-wide association studies (GWAS), the characteristics of eQTL and the relationship between different tissues, and between different eQTL types.

**Additional file 2: Table S1.** STAR parameters used for alignment.

**Additional file 3: Figure S2.** Data information. a: Distribution of imputed whole-genome single nucleotide polymorphisms (SNPs) in the sheep genome. Horizontal axis is the size of the chromosome, vertical axis is the chromosome number (from chromosome 1 to 26), the color scale denotes the number of SNPs within 1 Mb-windows. b: 3′/5′ bias. The plots of coverage for all expressed genes in liver (grey) and muscle (yellow) indicated little 5′ bias. Error bars represent the standard error of coverage for the 149 samples. c: Gene saturation in liver (grey) and muscle (yellow). Gene and splice junction saturation is reached when an increment in the number of reads does not result in additional expressed genes being detected or in more features, e.g., splice junctions, called. Error bars represent the standard error for the number of detected genes. d: Saturation of total splice junctions in liver (grey) and muscle (yellow). Error bars represent the standard error for the detected splice junctions. e: Saturation of annotated splice junctions in liver (grey) and muscle (yellow). f: Saturation of novel splice junctions in liver (grey) and muscle (yellow). Error bars represent the standard error for the detected splice junctions.

**Additional file 4: Table S2.** RNA-seq library information. Mean, minimum, maximum and median values of the number of read pairs that pass trimming and filtering (clean reads), and are uniquely mapped reads, and the proportion of clean reads that map uniquely to the genome in liver and muscle samples. The distribution of the proportion of clean reads that are uniquely mapped in liver and muscle samples is also shown.

**Additional file 5: Figure S3.** Circle Manhattan plot of expression quantitative trait loci (eQTL, which include gene expression QTL (geQTL); exon expression QTL (eeQTL) and splicing QTL (sQTL)) in liver (a) and muscle (b). From inside to outside, the circle Manhattan plot denotes geQTL, eeQTL and sQTL, respectively. Red dash line in each Manhattan plot represents the threshold (FDR < 0.01). Circle Manhattan plots were plotted using the CMplot R package (https://github.com/YinLiLin/R-CMplot).

**Additional file 6: Figure S4.** Overlap between liver and muscle for the three types of expression quantitative trait loci (eQTL, which include gene expression QTL (geQTL); exon expression QTL (eeQTL) and splicing QTL (sQTL)). a: Venn diagrams showing the expression quantitative trait loci detected in liver and in muscle, and in both. b: Gene expression, exon expression, and intron excision events with eQTL detected in liver and in muscle, and in both. c: Correlation of eQTL effects (t-value of eQTL) for which the eQTL were significant both in liver and muscle.

**Additional file 7: Table S3.** Significant expression quantitative trait loci (eQTL, which include gene expression QTL (geQTL), exon expression QTL (eeQTL), and splicing QTL (sQTL)) in liver and muscle overlapping with genome-wide association study (GWAS) hit regions linked with body composition [28]. This file contains six columns. Column 1: tissue type, namely, muscle or liver; column 2, eQTL type, namely, geQTL, eeQTL or sQTL; column 3: eQTL ID; column 4: eQTL FDR-value; column 5: GWAS SNP ID; column 6: GWAS FDR-values.

## Data Availability

Whole-genome sequence genotypes used for imputation are available at the European Variant Archive (https://www.ebi.ac.uk/eva/?eva-study=PRJEB31241). Raw RNA sequence read data are available at NCBI PRJNA689847. Processed datasets used and/or analyzed during the current study are available from the corresponding author on reasonable request. The datasets supporting the conclusions of this article are included in the article.
