# Supplementary figures and images for: Expression quantitative trait loci in sheep liver and muscle contribute to variations in meat traits
Source: Genet Sel Evol. 2021 Jan 18;53:8. doi: 10.1186/s12711-021-00602-9 (PMC7812657; doi:10.1186/s12711-021-00602-9)

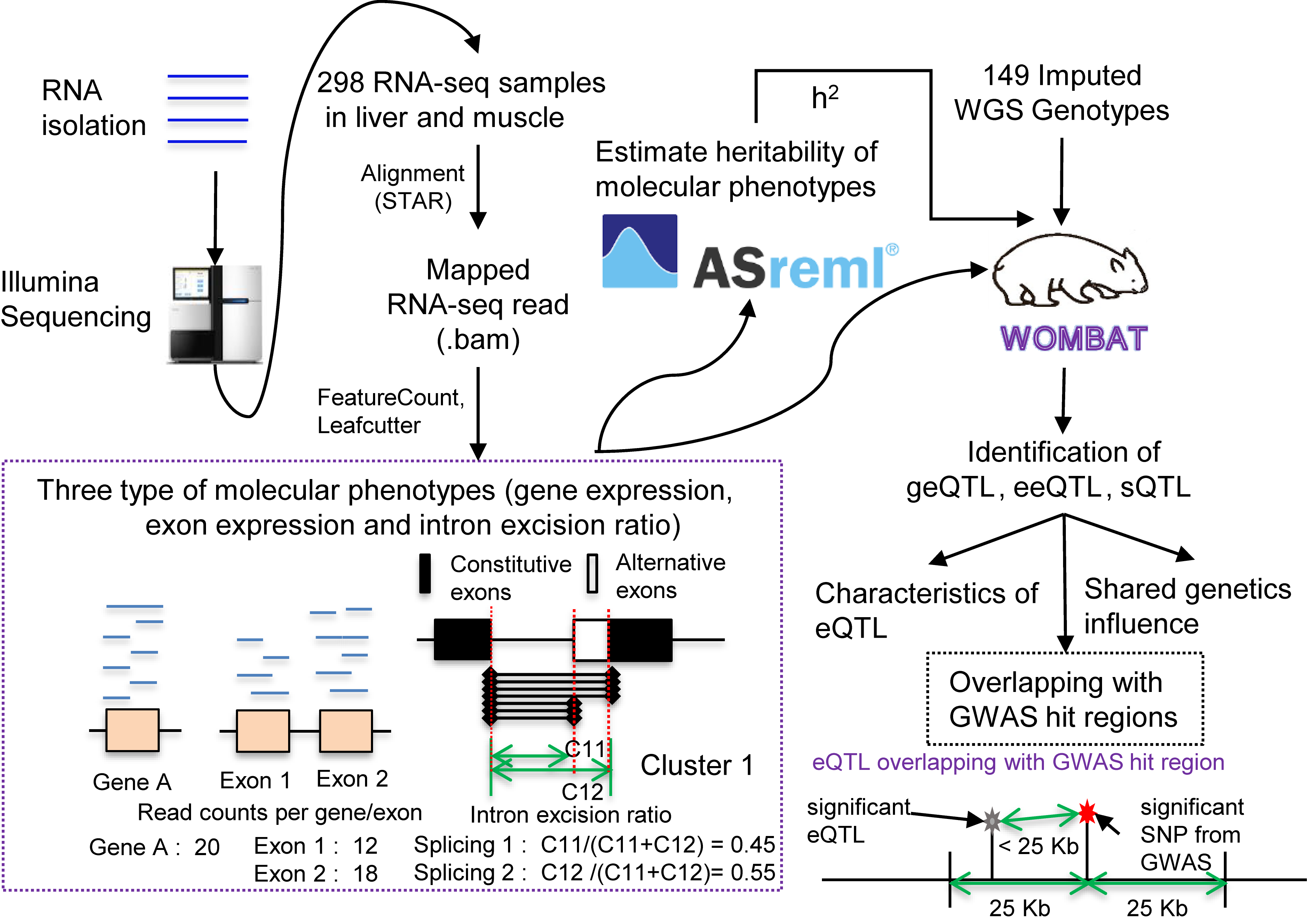

Supplement: Supplementary file 1 — Additional file 1: Figure S1. Overview of the analysis. In total, 298 RNA-seq data in liver and muscle from 149 crossbred male wether lambs were aligned to the sheep reference genome Oar_v3.1 (ftp://ftp.ensembl.org/pub/release-91/fasta/ovis_aries/dna/) using STAR along with the annotation file (Ovis_aries.Oar_v3.1.91.gtf.gz, containing 27,054 genes). Gene and exon expression levels were quantified by counting the reads of the gene and exon using FeatureCount. RNA-splicing was estimated by calculating intron excision ratio using Leafcutter. Heritability (h2) of the three molecular phenotypes (gene expression, exon expression and intron excision ratio) were estimated using ASreml®. Wombat software was used to identify cis expression quantitative trait loci (eQTL, which include gene expression QTL (geQTL); exon expression QTL (eeQTL) and splicing QTL(sQTL)) within 1 Mb of the gene, exon or intron excision event. We investigated the overlap between eQTL and two genome-wide association studies (GWAS), the characteristics of eQTL and the relationship between different tissues, and between different eQTL types. [file 12711_2021_602_MOESM1_ESM.tif]

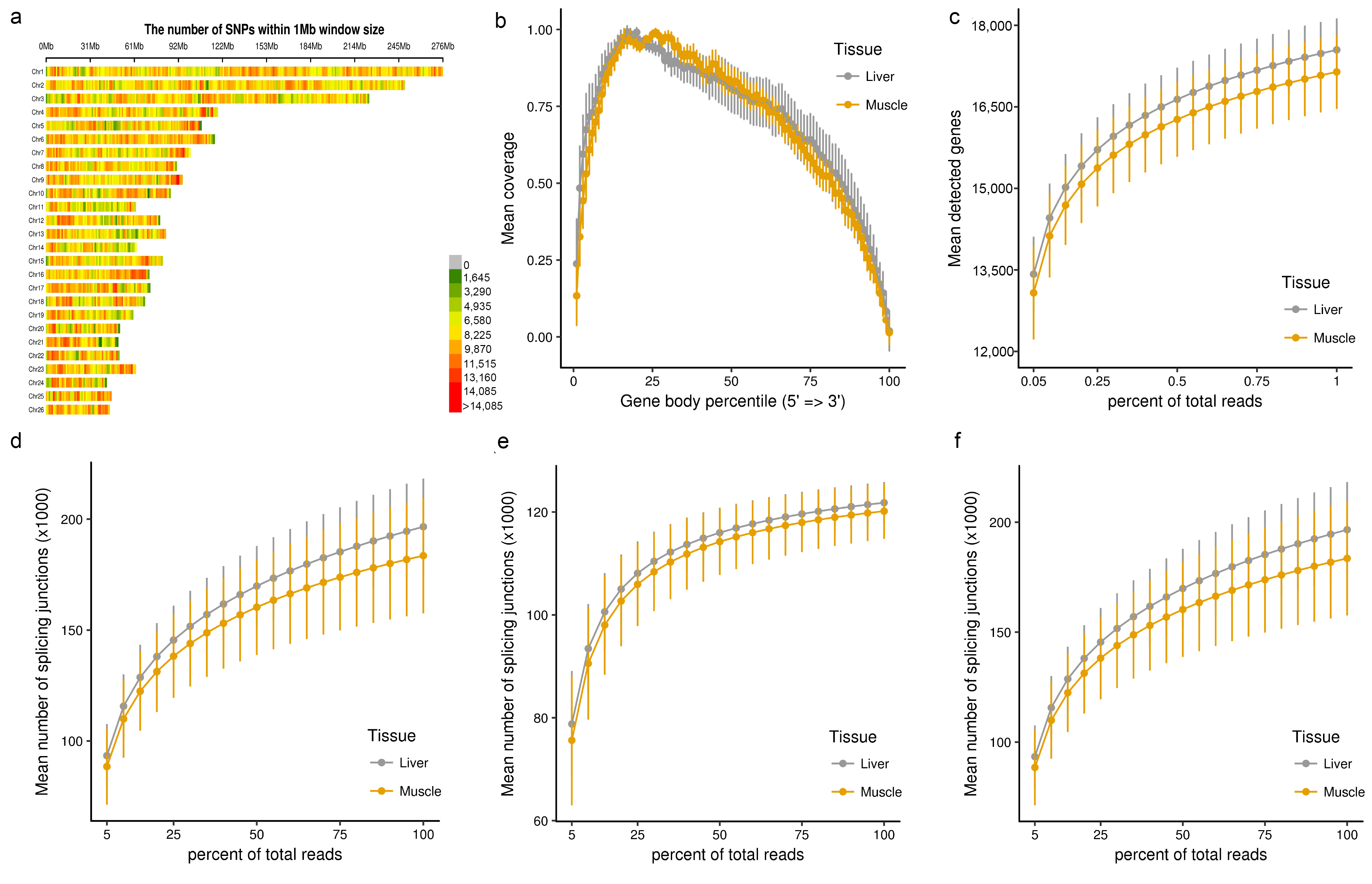

Supplement: Supplementary file 3 — Additional file 3: Figure S2. Data information. a: Distribution of imputed whole-genome single nucleotide polymorphisms (SNPs) in the sheep genome. Horizontal axis is the size of the chromosome, vertical axis is the chromosome number (from chromosome 1 to 26), the color scale denotes the number of SNPs within 1 Mb-windows. b: 3′/5′ bias. The plots of coverage for all expressed genes in liver (grey) and muscle (yellow) indicated little 5′ bias. Error bars represent the standard error of coverage for the 149 samples. c: Gene saturation in liver (grey) and muscle (yellow). Gene and splice junction saturation is reached when an increment in the number of reads does not result in additional expressed genes being detected or in more features, e.g., splice junctions, called. Error bars represent the standard error for the number of detected genes. d: Saturation of total splice junctions in liver (grey) and muscle (yellow). Error bars represent the standard error for the detected splice junctions. e: Saturation of annotated splice junctions in liver (grey) and muscle (yellow). f: Saturation of novel splice junctions in liver (grey) and muscle (yellow). Error bars represent the standard error for the detected splice junctions. [file 12711_2021_602_MOESM3_ESM.tif]

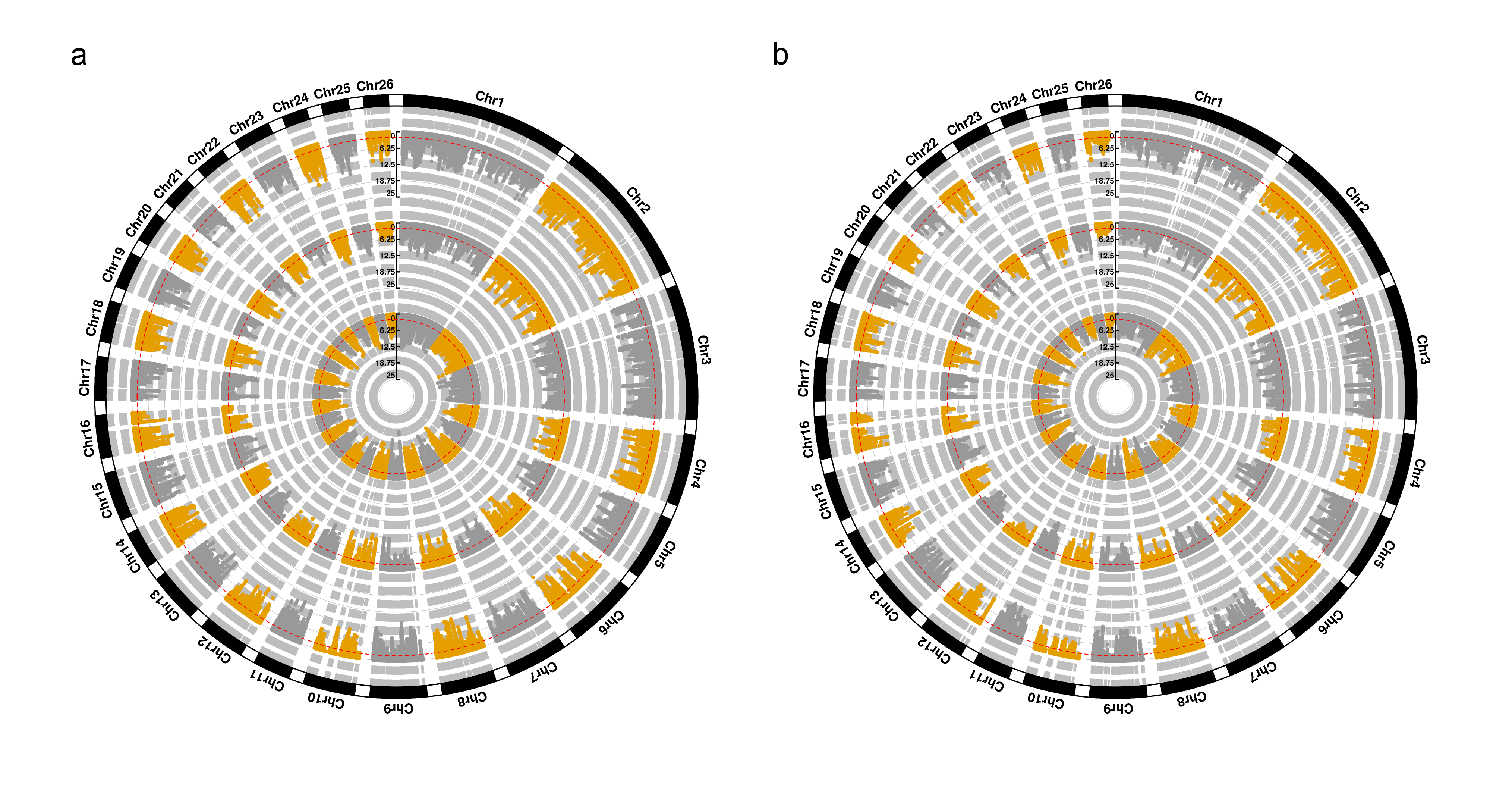

Supplement: Supplementary file 5 — Additional file 5: Figure S3. Circle Manhattan plot of expression quantitative trait loci (eQTL, which include gene expression QTL (geQTL); exon expression QTL (eeQTL) and splicing QTL (sQTL)) in liver (a) and muscle (b). From inside to outside, the circle Manhattan plot denotes geQTL, eeQTL and sQTL, respectively. Red dash line in each Manhattan plot represents the threshold (FDR < 0.01). Circle Manhattan plots were plotted using the CMplot R package (https://github.com/YinLiLin/R-CMplot). [file 12711_2021_602_MOESM5_ESM.tif]

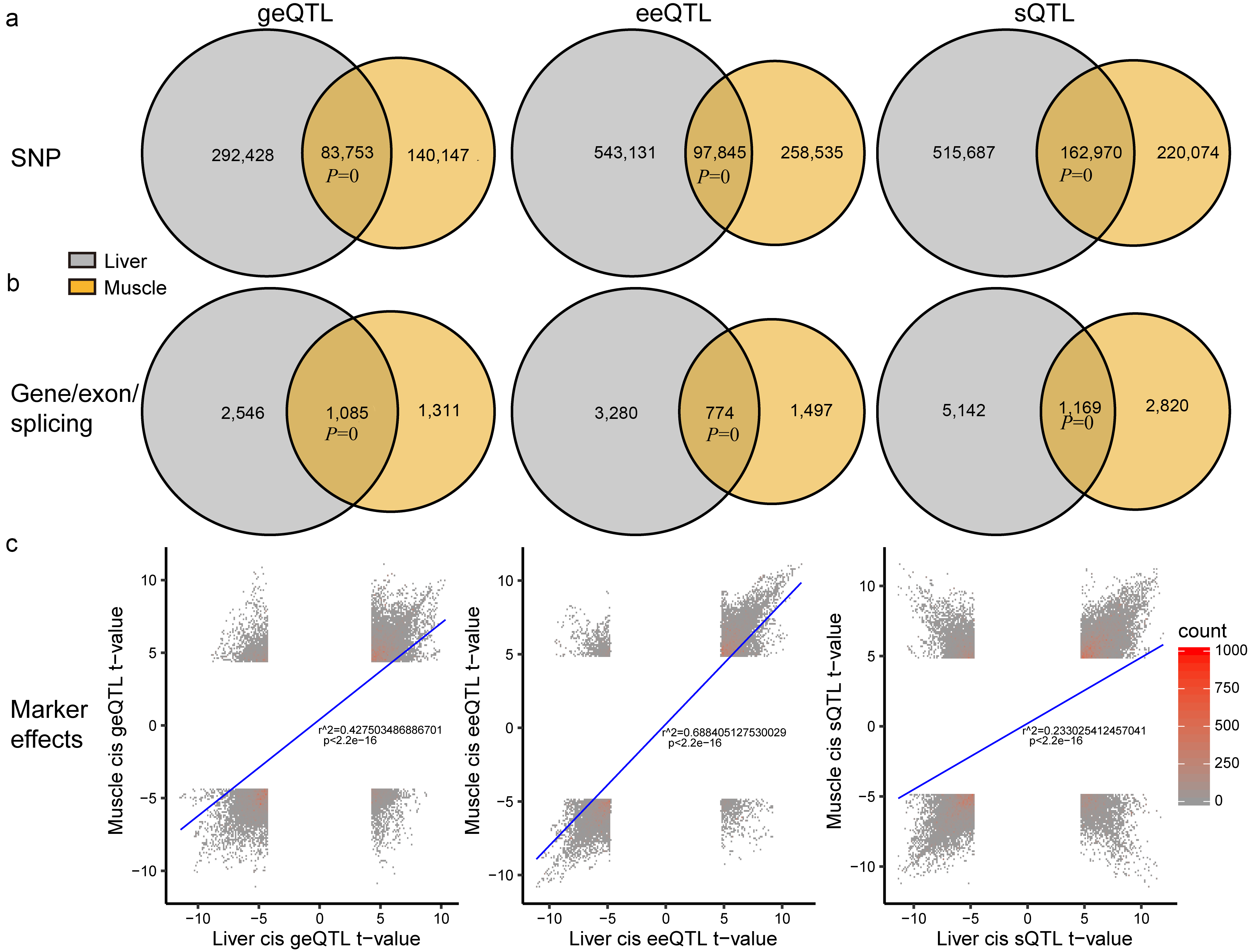

Supplement: Supplementary file 6 — Additional file 6: Figure S4. Overlap between liver and muscle for the three types of expression quantitative trait loci (eQTL, which include gene expression QTL (geQTL); exon expression QTL (eeQTL) and splicing QTL (sQTL)). a: Venn diagrams showing the expression quantitative trait loci detected in liver and in muscle, and in both. b: Gene expression, exon expression, and intron excision events with eQTL detected in liver and in muscle, and in both. c: Correlation of eQTL effects (t-value of eQTL) for which the eQTL were significant both in liver and muscle. [file 12711_2021_602_MOESM6_ESM.tif]
