# Supplementary material for: Expression quantitative trait loci in sheep liver and muscle contribute to variations in meat traits
Source: Genet Sel Evol. 2021 Jan 18;53:8. doi: 10.1186/s12711-021-00602-9 (PMC7812657; doi:10.1186/s12711-021-00602-9)
Supplement: Supplementary file 2 — Additional file 2: Table S1. STAR parameters used for alignment. [file 12711_2021_602_MOESM2_ESM.docx]

| 01  02  03  04  05  06  07  08  09  10  11  12  13  14  15  16  17  18 | ${binDIR}STAR --runMode alignReads \  --outFileNamePrefix ${animalID} \  --runThreadN 8 \  --genomeDir ${genomeDir} \  --readFilesIn ${dataDir}${animalID}.R1.fastq ${data}${animalID}.R2.fastq \  --limitOutSJcollapsed 1000000 \  --limitSjdbInsertNsj 1000000 \  --outFilterMultimapNmax 100 \  --outFilterMismatchNmax 33 \  --outFilterMismatchNoverLmax 0.3 \  --seedSearchStartLmax 12 \  --alignSJoverhangMin 15 \  --outFilterMatchNminOverLread 0 \  --outFilterScoreMinOverLread 0.3 \  --winAnchorMultimapNmax 50 \  --alignSJDBoverhangMin 3 \  --outFilterType BySJout \  --outSAMunmapped Within |
| --- | --- |

**Table S1 STAR parameters used for alignment RNA-seq data to sheep reference genome**
