# Supplementary material for: Expression quantitative trait loci in sheep liver and muscle contribute to variations in meat traits
Source: Genet Sel Evol. 2021 Jan 18;53:8. doi: 10.1186/s12711-021-00602-9 (PMC7812657; doi:10.1186/s12711-021-00602-9)
Supplement: Supplementary file 4 — Additional file 4: Table S2. RNA-seq library information. Mean, minimum, maximum and median values of the number of read pairs that pass trimming and filtering (clean reads), and are uniquely mapped reads, and the proportion of clean reads that map uniquely to the genome in liver and muscle samples. The distribution of the proportion of clean reads that are uniquely mapped in liver and muscle samples is also shown. [file 12711_2021_602_MOESM4_ESM.doc]

| Tissue | Value type | Clean read pairs | Uniquely mapped read pairs | Uniquely mapped rate (%) | Distribution of uniquely mapped rate in liver and muscle |
| --- | --- | --- | --- | --- | --- |
| Liver | Mean value | 27,779,864 | 24,905,476 | 89.78 | 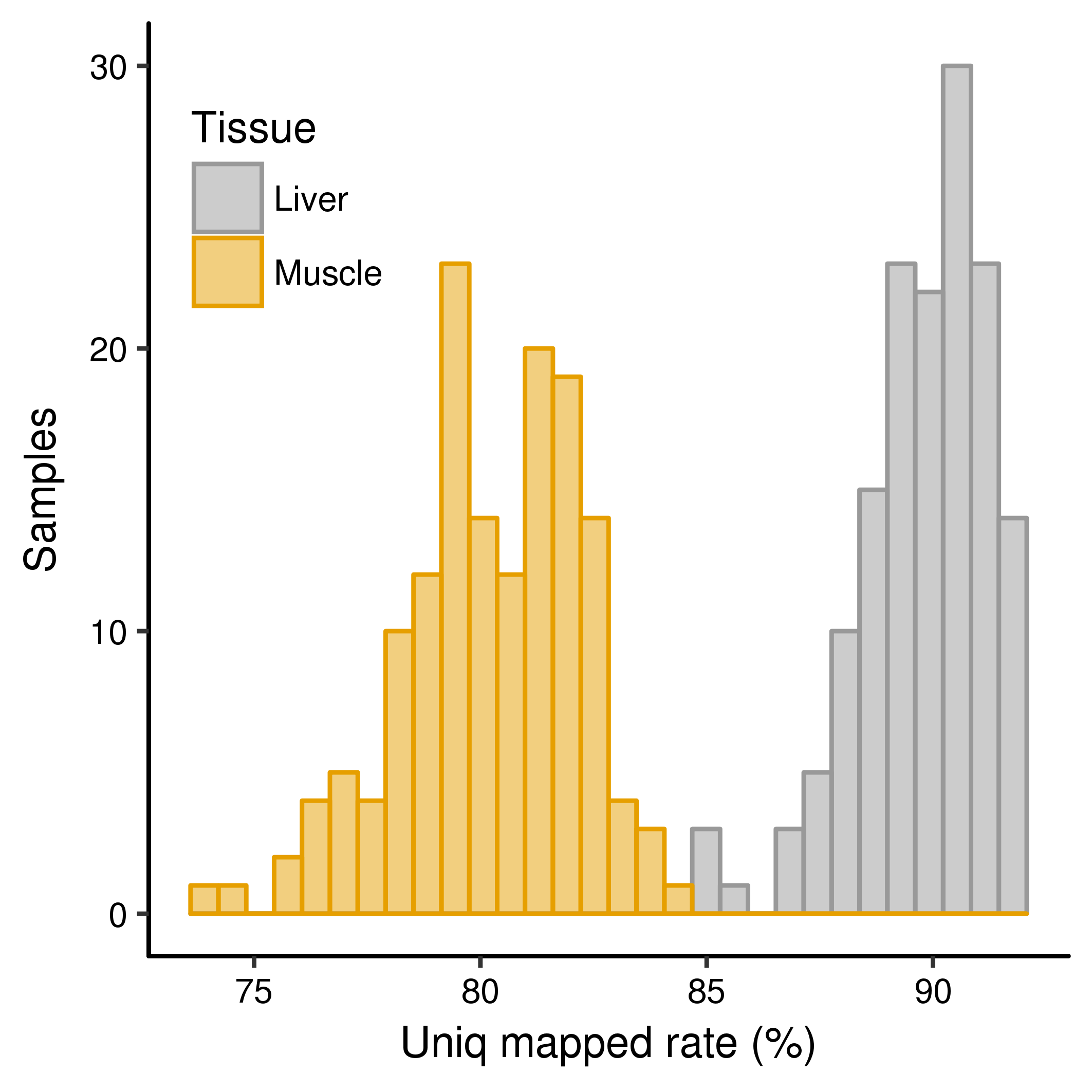 |
|  | Min value | 9,486,361 | 8,331,440 | 84.80 |  |
|  | Max value | 109,029,082 | 92,669,403 | 92.02 |  |
|  | Median | 25,540,057 | 23,108,754 | 90.05 |  |
| Muscle | Mean value | 28,457,517 | 22,790,783 | 80.20 |  |
|  | Min value | 8,376,884 | 6,717,817 | 74.16 |  |
|  | Max value | 151,898,979 | 122,555,852 | 84.46 |  |
|  | Median | 23,150,888 | 18,298,635 | 80.24 |  |

**Table S2 RNA-seq data information of liver and muscle**
